# Ex Vivo Drug Susceptibility of *Plasmodium malariae* Isolates to Antimalarial Drugs in Gabon

**DOI:** 10.3390/pathogens14050453

**Published:** 2025-05-06

**Authors:** Yudi T. Pinilla, Anton Hoffmann, Maxim Viehweg, Nathanaël Saison, Stravensky Terence Boussougou Sambe, Ange Gatien Doumba Ndalembouly, Barclaye Ngossanga, Florence Awamu, Ayola Akim Adegnika, Steffen Borrmann

**Affiliations:** 1 Centre de Recherches Médicales de Lambaréné, Lambaréné BP 242, Gabon; anton.hoffmann95@outlook.de (A.H.); maxim.viehweg@student.uni-tuebingen.de (M.V.); nat.saison@gmail.com (N.S.); stravsambe2@gmail.com (S.T.B.S.); angegatien@gmail.com (A.G.D.N.); barclayengossanga@gmail.com (B.N.); aadegnika@cermel.org (A.A.A.); steffen.borrmann@uni-tuebingen.de (S.B.); 2 Institute for Tropical Medicine, University of Tübingen, 72074 Tübingen, Germany; 3 German Center for Infection Research, 72074 Tübingen, Germany

**Keywords:** *P. malariae*, drug susceptibility assay, malaria, Gabon

## Abstract

*Plasmodium malariae* is a neglected human malaria parasite despite its global distribution and propensity for persistent, sub-microscopic infections, which are associated with a mild but significant disease burden. Artemisinin-based therapies appear to be efficacious, but the susceptibility profiles of field isolates are largely unknown. We performed ex vivo assays with isolates collected from asymptomatic volunteers in Gabon. The mean concentrations required to inhibit 50% of growth (IC50) with chloroquine (n = 21), artesunate (n = 20), atovaquone (n = 21), and lumefantrine (n = 14) were 7.2 nM, 2.7 nM, 3.1 nM, and 7.4 nM, respectively. Our study provides novel data on the ex vivo susceptibility of *P. malariae* to several key antimalarials, including the first dataset for atovaquone.

## 1. Introduction

*Plasmodium malariae* is a neglected human malaria parasite, occurring often co-endemic with *Plasmodium falciparum* in regions such as sub-Saharan Africa, South America, Indonesia, Southeast Asia, and the Western Pacific [1]. Unlike *P. falciparum*, *P. malariae* has a longer, 72 h intraerythrocytic replication cycle, resulting in highly periodic fever peaks in non-immune patients, known as quartan malaria. The parasite is also known for its ability to cause chronic infections, which can persist and recur after years or even decades after the initial infection [2,3]. While *P. malariae* infections are generally far less pathogenic than those caused by *P. falciparum*, repeated and prolonged exposure can lead to anemia, contributing to the disease burden in affected populations [4].

Recent evidence suggests that *P. malariae* has become more prevalent in areas with low *P. falciparum* transmission following successful intervention efforts [5]. Sub-microscopic and thus, largely undiagnosed and untreated *P. malariae* infections constitute a significant challenge to malaria elimination efforts [6,7]. Whereas, microscopic-based surveys typically detect *P. malariae* infections in 1.3% to 5% of individuals [7,8], more sensitive molecular techniques, such as PCR or qPCR, reveal significantly higher prevalence rates of up to 30% and 10% in asymptomatic and symptomatic individuals, respectively [8,9,10]. Despite these findings, the actual burden of *P. malariae* remains poorly understood, largely due to the absence of routine diagnostic testing and the limited number of systematic epidemiological studies [1].

The World Health Organization (WHO) recommends artemisinin-based combination therapy (ACT) as the primary treatment for *P. falciparum*. However, there are currently no established treatment guidelines for *P. malariae*, and limited studies have assessed the efficacy of ACTs against this parasite [11,12,13]. In recent years, ACT resistance has emerged in Southeast Asia and a potential spread to other malaria-endemic regions jeopardizes global malaria control efforts [14]. In contrast to *P. falciparum* and *P. vivax*, evidence of chloroquine resistance in *P. malariae* remains limited [15]. However, more recently, treatment failures of artemether-lumefantrine for *P. malariae* malaria have been reported [16,17]. This highlights the need for further research to evaluate the in vivo and *in vitro* efficacy of these drugs. Due to the absence of continuous in vitro cultivation methods for *P. malariae*, ex vivo drug assays are currently the only option for gathering data on the drug susceptibility of this parasite. This study aims to evaluate the susceptibility of commonly used antimalarial drugs, including artesunate (AS), atovaquone (ATQ), lumefantrine (LUM), and chloroquine (CQ), against *P. malariae*.

## 2. Materials and Methods

### 2.1. Sample Collection

The study was conducted as a part of the CoMal project (DFG BO 2494/3-1), a cross-sectional prevalence study of non-falciparum malaria species in rural communities of the Moyen-Ogooué province of Gabon. Between January and April 2020, and again from April to August 2022, asymptomatic volunteers older than 1 year were screened for malaria infection by obtaining finger-prick blood samples. Thick blood smears were stained with 10% Giemsa solution for 45 min and examined microscopically by two experienced microscopists at 1000× magnification. Slide readings and calculations of the parasitemia were performed according to the Lambaréné method [18]. Only individuals with microscopic evidence of *P. malariae* infections (either as a mono-infection or mixed infection with *P. falciparum* or *P. ovale*), a hemoglobin concentration of ≥6 g/dL, and who had not taken antimalarial treatment within 3 months of sample collection were included. After obtaining written informed consent, blood samples were collected via venipuncture into sodium heparin vacutainers: 10 mL from participants aged ≥ 18 years and 5 mL from children > 5 years. Following sample collection, all patients were treated with artemether-lumefantrine (Coartem®), in accordance with the national treatment guidelines.

### 2.2. Ex Vivo Susceptibility Test

The ex vivo susceptibility of *P. malariae* isolates was assessed using a schizont maturation assay [19]. Fresh blood samples were centrifuged at 2000× *g* for 5 min at 37 °C, and the resulting red blood cell pellet was washed three times with incomplete culture medium (RPMI-1640 supplemented with 25 mM HEPES, 25 mM NaHCO_3_). Erythrocyte pellets were then resuspended in complete culture medium (RPMI-1640 supplemented with 25 mM HEPES, 25 mM NaHCO_3_, 20 mg/L hypoxanthine, 2.4 mM L-glutamine, 10 mg/L gentamycin, 5 g/L AlbuMAX^™^ II and 20% human AB serum), adjusted to a final hematocrit of 4%. The parasitized cells were distributed in 96-well drug plates, which were pre-dosed with 9 serial concentrations of CQ (2000 to 0.95 nM), AS (100 to 0.26 nM), ATQ (100 to 0.26 nM), and LUM 250 to 0.78 nM. CQ, AS, ATQ, and LUM were purchased from Sigma (Tokyo, Japan). Stock solutions of CQ (10 mM), AS (10 mM), ATQ (10 mM), were prepared in DMSO and LUM (10 mM) in RPMI medium. Each drug concentration was tested in triplicate.

Fifty microliters of the Blood Medium Mixture (BMM) was then added to each well. After the parasitized blood samples were added, the plates were incubated at 37 °C for up to 48 h in a multi-gas incubator (5% CO_2_, 5% O_2_, and 90% N_2_ with 80% humidity). Incubation was stopped when >50% of ring-stage parasites had differentiated to schizonts in the drug-free control wells. Schizont count per 100 parasites was determined by microscopy, with schizonts classified only when at least 4 merozoites per cell were discernable. The IC50 values were determined using nonlinear regression and a three-parameter dose–response curve. The stock drug solutions were validated using NF54 *P. falciparum* strain.

### 2.3. Real-Time qPCR Assay

An established qPCR assay was used to confirm *P. malariae* mono- or co-infected fresh isolates. A 500 µL sample of infected blood was mixed in a 1:2 ratio with DNA/RNA shield and RNA was extracted using the Microprep Plus Kit D7005 (Zymo Research, Irvine, CA, USA). The real-time qPCR was performed at the Institute for Tropical Medicine, Tübingen, Germany, following a previously described protocol [20]. Total RNA extraction was performed using the Quick-DNA/RNA™ Microprep Plus Kit (Zymo Research, Irvine, CA, USA) according to the manufacturer’s instructions. Purified RNA samples were immediately stored at −20 °C until use.

qPCR assays were conducted in parallel to detect *P. malariae* with the highest sensitivity, particularly in case of co-infections with *P. falciparum* or other *Plasmodium* species (LightCycler 480, Roche, Mannheim, Germany). The first assay identified *P. malariae* using primers that amplify a 100 bp fragment of the small-subunit rRNA gene (18 S), which is conserved across human malaria pathogens [21]. Each reaction required 1 µL RNA template, 5 µL SensiFAST™ One-Step Mix (Bioline, London, UK), 0.1 µL transkriptase, 0.2 µL 10 μM TaqMan probe, 0.4 µL of each 10 μM primer, and 2.9 µL water (10 µL total reaction volume).

The second assay differentiated between *P. falciparum* and other *Plasmodium* species, including *P. malariae*, *P. vivax*, and *P. ovale*, as described previously [21]. Primers and probes were designed using OligoArchitect online (Sigma-Aldrich, Tokyo, Japan), based on 18 S rRNA gene sequences from GenBank. Each reaction consisted of 1 µL RNA template, 5 µL SensiFAST™ One-Step Mix (Bioline, London, UK), 0.1 µTranscriptase, 0.4 µL of each 10 μM TaqMan probe, 0.8 µL of each 10 μM primer, and 1.5 µL water. After an initial transcription step of 20 min at 45 °C, followed by a hot-start step of 5 min at 95 °C, the cycling conditions were identical for both assays, allowing simultaneous runs in a single analysis step. The cycling conditions included 5 min at 95 °C, followed by 50 cycles of 93 °C for 15 s and 60 °C for 60 s. All samples were analyzed in triplicate, and each assay included a non-template control and a positive control in triplicates, respectively.

### 2.4. Statistical Analysis

Dose−response curves and 50% inhibitory concentrations (IC50) of the drugs of the clinical isolates were calculated by nonlinear regression analysis using GraphPad Prism (version 9) software (GraphPad Software Inc., San Diego, CA, USA). The type of distribution was evaluated using a Shapiro–Wilk or Kolmogorov–Smirnov test. Differences in drug susceptibility between drugs and *P. falciparum* and *P. malariae* were determined using a non-parametric Mann–Whitney test. A *p*-value < 0.05 was considered as significant. All statistical analyses were performed using GraphPad Prism (version 9) software (GraphPad Software Inc., San Diego, CA, USA).

The output of ultrasensitive qPCR reactions was examined by visual inspection and the cycle threshold (Ct) was calculated using LightCycler 480 Software (version 1.5.1.62) via the second derivative maximum method. A Ct value below the threshold of 40 indicated a positive sample.

## 3. Results

Between January and April 2020 and April and August 2022, a total of 1303 asymptomatic participants were screened for malaria. Of these, 42 (3.2%) participants were found to be infected with *P. malariae* by microscopy. Among the 42 infected participants, 24 (57%) had a *P. malariae* mono-infection, while 18 (43%) were co-infected with *P. malariae* and *P. falciparum*. Of the 42 *P. malariae* isolates collected, only 21 yielded ex vivo assay results. To confirm *P. malariae* mono-infection and co-infection with *P. falciparum* in the 21 isolates tested, qPCR was performed. The qPCR analyses showed that 9 out of the 21 isolates were *P. malariae* mono-infected (Table 1). The parasitemia of isolates ranged from 153 to 8620 parasites/µL, with a median assay duration of 36 h (range: 13 to 48 h). For the drug sensitivity analysis, only isolates with ≥50% ring-stage parasites at the start of the assay were included. Some drugs, such as AS and LUM, were not tested on certain isolates due to the insufficient blood volume collected.

**Figure 1:**
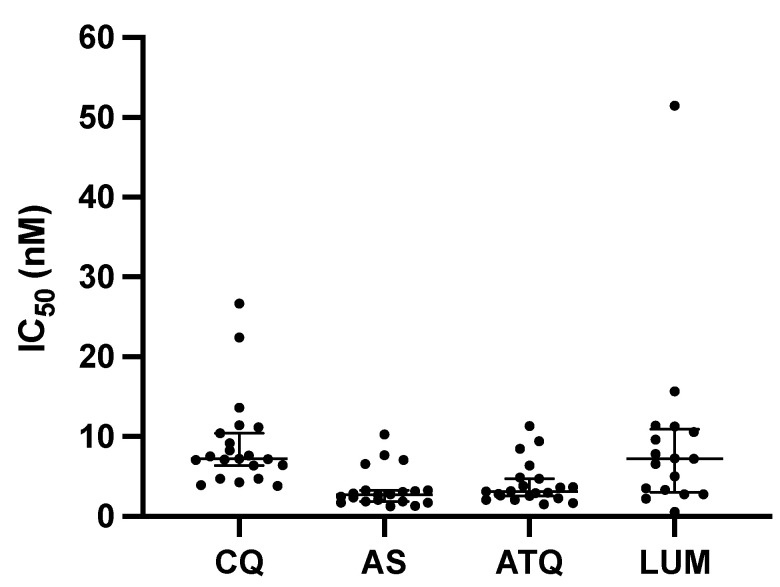
Distribution of IC50 values of *P. malariae* isolates for each drug (CQ, AS, ATQ, and LUM). CQ, chloroquine; AS, artesunate; ATQ, atovaquone; LUM, lumefantrine. Each data point for a particular drug shows mean IC50 values. Error bars indicate medians and 95% confidence intervals.

IC50 values for the antimalarial drugs were determined for each isolate (Table 2 and Figure 1). Stock drug solutions were validated using the fully sensitive, laboratory-adapted *P. falciparum* NF54 strain, yielding consistently low nanomolar IC50 values (Table 2). The median ex vivo IC50 values for each drug were as follows: CQ (n = 21, IC50 = 7.2 nM, range: 3.8–26.7 nM), AS (n = 20, IC50 = 2.7 nM, range: 1.3–10.2 nM), ATQ (n = 21, IC50 = 3.1 nM, range: 1.5–11.3 nM), and LUM (n = 14, IC50 = 7.4 nM, range: 0.6–51.5 nM). All IC50s for chloroquine were below 35 nM, suggesting that *P. malariae* isolates from Gabon remain susceptible to this drug. 

## 4. Discussion

Drug susceptibility profiles are well documented for *P. falciparum* and *P. vivax*, but there is limited knowledge regarding the activity of antimalarials against *P. malariae* isolates [22,23]. The high prevalence of sub-microscopic *P. malariae* infections in sub-Saharan African countries, along with its co-endemicity with *P. falciparum*, underscores the importance of understanding its drug susceptibility profile to inform effective treatment and elimination strategies. While in vitro and ex vivo cultivation of *P. falciparum* has facilitated extensive research and the development of effective drug treatments, the absence of similar cultivation systems for the asexual blood stages of *P. malariae* presents significant challenges to monitor existing antimalarial treatment strategies and validate novel antimalarial drug candidates [24]. In this study, we assessed the ex vivo susceptibility of *P. malariae* to four commonly used antimalarial drugs, chloroquine (CQ), artesunate (AS), atovaquone (ATQ), and lumefantrine (LUM), by testing freshly collected isolates.

The results showed generally low IC50 values for all tested drugs, with ATQ and AS displaying the highest potency, suggesting the continued susceptibility of *P. malariae* to these compounds. Our findings are consistent with those reported in earlier clinical studies on *P. falciparum* and *P. malariae* in Gabon [11,21,25]. Importantly, all of the CQ IC50 values were below the commonly referenced resistance thresholds [15], reinforcing the notion that *P. malariae* remains susceptible to CQ in Gabon. However, several limitations should be considered when interpreting our findings. Although 42 *P. malariae* infections were found in the study, the ex vivo susceptibility testing was successful in only 21 *P. malariae* isolates. This relatively small sample size limits the statistical power and generalizability of the results. Additionally, the study was conducted in a single region (Gabon), and the isolates cannot represent the genetic and phenotypic diversity of *P. malariae* found in other parts of sub-Saharan Africa or globally [26]. Larger, multi-country studies incorporating isolates from diverse transmission settings, including West, Central, and East Africa, as well as Southeast Asia and South America, are necessary to draw more robust data on the drug susceptibility of *P. malariae*.

A recent study from West Africa (Mali) has identified unusually high IC50 values for CQ, LUM and artemether against *P. malariae* isolates [22]. Although most clinical trials involving artemisinin derivatives or ACTs report sustained high efficacy [11,13], there have been a few instances of treatment failures in *P. malariae* infections [13,15,16]. The longer erythrocytic cycle of *P. malariae* may contribute to a natural refractoriness to 3-day ACT regimens, which may explain the persistence of infections, i.e., treatment failure, when using sensitive PCR during follow-up [16]. On the other hand, the survival of wild-type *P. malariae* parasites could potentially translate to lower selection pressure for mutants tolerant or resistant to partner drugs with short to medium elimination half-lives. More recently, a population-genomic analysis of global *P. malariae* isolates has identified mutations in PmDHFR that correspond to variants in PfDHFR conferring resistance to the slowly eliminated pyrimethamine [26], which we did not test in this study. The drug-resistant phenotype of these PmDHFR mutations were subsequently confirmed by ortholog replacement experiments in *P. knowlesi* [26]. 

Our study is the first to report the ex vivo sensitivity of *P. malariae* to ATQ. The results indicate that ATQ effectively inhibits *P. malariae* isolates, with IC50 values comparable to those reported for *P. falciparum* [27]. Atovaquone-proguanil is a highly effective non-ACT drug for the treatment of multidrug-resistant *P. falciparum*, and it is considered the drug of choice for both chemoprophylaxis in non-immune travelers to regions with reported multidrug resistance and the treatment of imported cases of acute uncomplicated *P. falciparum* infections in non-endemic countries. Given the frequent occurrence of *P. malariae* in Central and West Africa, often in mixed infections with *P. falciparum*, these findings underscore the complexity of malaria treatment in regions where species identification is difficult. Our findings support the continued use of combinations containing ATQ and LUM as effective oral regimens in case of diagnostic uncertainty or presumptive treatment of acute, uncomplicated malaria. This is particularly relevant in areas where mixed-species infections are prevalent or where limitations in diagnostic tool uncertainty limits species-specific treatment strategies.

Our results showed some variability in the IC50 values for LUM (ranging from 0.6 to 51.5 nM; median 7.2; interquartile range 3.0–10.9), mainly due to a single outlier (isolate #627). The observed variation may reflect phenotypic plasticity among the *P. malariae* isolates, potentially due to genetic variation. Nonetheless, technical and methodological factors could also have contributed to this variability. For instance, differences in the incubation duration in the ex vivo assay may have influenced schizont maturation and, consequently, the calculated IC50 values. Although only isolates with ≥50% ring-stage parasites were tested, some variability in parasite synchronization and maturation likely occurred, particularly in those samples with prolonged or shortened incubation times. While most assays were completed after 36 h, some were stopped earlier or later (e.g., isolate 1077 was incubated for 45 h, while isolate 1179 was only incubated for 13 h). The fact that 11 out of the 21 microscopically determined mono-infected *P. malariae* isolates were later shown by qPCR to be co-infected with *P. falciparum* could have also affected our results (such as in the case of isolate #627). In addition, and when compared to drugs like chloroquine (CQ) or artesunate (AS), suboptimal LUM solubility or inconsistencies in drug plate preparation could have affected the effective drug concentration, thereby impacting IC50 measurements. Without accompanying molecular data or repeat assays, it remains unclear whether this variability represents emerging tolerance or is simply an artifact of the assay conditions. Further investigation, including the genotyping of known drug resistance markers, is necessary to clarify this hypothesis. In addition, ex vivo response rates to lumefantrine in *P. falciparum* have also shown considerable variability [28,29].

In summary, our findings show that *P. malariae* isolates from Gabon remain susceptible to CQ, AS, ATQ, and LUM in an ex vivo assay. While these results are promising, a larger number of isolates, particularly from diverse geographic regions, are needed to provide a comprehensive understanding for guiding malaria control strategies. Diagnosing *P. malariae* infections remains challenging, particularly due to low parasite densities and the frequent occurrence of co-infections with other *Plasmodium* species. For prophylactic treatment, the WHO recommends drug combinations used in seasonal malaria chemoprevention in certain African settings; however, their effectiveness against *P. malariae* requires further study. Additionally, a better understanding of the impact of existing and emerging genetic variation on drug sensitivity of *P. malariae* is essential for developing effective, antimalarial-based elimination strategies.

## Figures and Tables

**Table 1:** Characteristics of the *P. malariae* isolates used for the susceptibility drug assay. **ND: Not determined**.

Isolate No.	Parasite Count, No. Parasites/µL	*Plasmodium* Species Microscopy	*Plasmodium* Species qPCR
12	153	P.m	P.m
27	2610	P.m-P.f	P.m-P.f
148	8620	P.m-P.f	P.m-P.f
149	1013	P.m-P.f	P.m-P.f
167	677	P.m	P.m
268	831	P.m-P.f	P.m-P.f
269	503	P.m-P.f	P.m-P.f
325	1237	P.m	P.m
326	773	P.m	P.m
392	2960	P.m	P.m
589	677	P.m	P.m-P.f
627	2175	P.m	P.m-P.f
678	1856	P.m-P.f	P.m-P.f
742	2436	P.m	P.m
1037	803	P.m-P.f	P.m-P.f
1063	387	P.m	P.m
1077	ND	P.m-P.f	ND
1179	462	P.m	P.m-P.f
1194	406	P.m	P.m
1320	715	P.m	P.m
1351	358	P.m	P.m-P.f

**Table 2:** IC50 values (nM) for CQ, AS, ATQ, and LUM of all individual isolates as well as the NF54 control strain. NA: Not available. No IC50 data are available for these compounds.

Isolate No.	CQ (nM)	AS (nM)	ATQ (nM)	LUM (nM)	Incubation Time (h)
12	7.14	1.73	11.33	2.76	36
27	3.93	2.09	3.60	2.20	36
148	9.20	7.68	6.37	6.60	36
149	22.45	2.90	3.81	3.52	36
167	6.39	7.10	3.69	7.24	36
268	7.20	6.55	4.88	7.85	36
269	3.83	2.36	3.17	9.63	36
325	7.51	2.76	2.58	10.59	36
326	7.64	1.70	2.83	5.05	36
392	4.29	2.48	2.65	11.37	36
589	26.67	3.09	8.46	15.68	36
627	7.18	2.69	2.95	51.48	36
678	10.41	10.26	9.45	11.26	36
742	7.10	NA	4.72	7.28	36
1037	4.74	1.29	1.52	3.32	36
1063	8.29	1.90	2.93	2.77	36
1077	11.17	3.30	2.09	NA	45
1179	6.40	1.85	2.29	NA	13
1194	11.44	3.30	2.09	NA	24
1320	4.74	1.32	1.68	0.59	36
1351	13.60	3.17	3.11	NA	48
NF54	5.81	3.03	4.44	3.57	24

## Data Availability

The data presented in this research are available in the manuscript.

## Abbreviations

The following abbreviations are used in this manuscript:

PCR	Polymerase Chain Reaction
WHO	World Health Organization
ACT	Artemisinin-based combination therapy
AS	Artesunate
ATQ	Atovaquone
LUM	Lumefantrine
CQ	Chloroquine
DNA	Deoxyribonucleic Acid
RNA	Ribonucleic Acid
IC50	Half-maximal inhibitory concentration
ATPG	Atovaquone-proguanil
BMM	Blood Medium Mixture
